# High-Throughput Sequencing-Based Immune Repertoire Study during Infectious Disease

**DOI:** 10.3389/fimmu.2016.00336

**Published:** 2016-08-31

**Authors:** Dongni Hou, Cuicui Chen, Eric John Seely, Shujing Chen, Yuanlin Song

**Affiliations:** ^1^Department of Pulmonary Medicine, Zhongshan Hospital, Fudan University, Shanghai, China; ^2^Department of Medicine, Division of Pulmonary and Critical Care Medicine, University of California San Francisco, San Francisco, CA, USA

**Keywords:** immune repertoire, high-throughput sequencing, infection, lymphocyte, bioinformatics

## Abstract

The selectivity of the adaptive immune response is based on the enormous diversity of T and B cell antigen-specific receptors. The immune repertoire, the collection of T and B cells with functional diversity in the circulatory system at any given time, is dynamic and reflects the essence of immune selectivity. In this article, we review the recent advances in immune repertoire study of infectious diseases, which were achieved by traditional techniques and high-throughput sequencing (HTS) techniques. HTS techniques enable the determination of complementary regions of lymphocyte receptors with unprecedented efficiency and scale. This progress in methodology enhances the understanding of immunologic changes during pathogen challenge and also provides a basis for further development of novel diagnostic markers, immunotherapies, and vaccines.

## Introduction

The adaptive immune system is composed of B and T cells that form a highly selective guard against evolving pathogens. The foundation of the adaptive immune response is based on the enormous diversity of T and B cell antigen receptors that can recognize epitopes from a near infinite number of different internal and external antigens. This profound diversity of T (TCRs) and B cell receptors (BCRs) is generated by V–D–J gene recombination of the TCR/BCR locus and subsequent somatic hypermutation and class-switching recombination of B cells after antigen stimulation. Thus, study of the immune repertoire, portrayed as the antigen-specific information within lymphocytes, has been a key to understanding the response of adaptive immunity during infection.

Despite extensive efforts using traditional techniques, analysis of the immune repertoire with high resolution has remained difficult. Several sequencing strategies, for example, Sanger sequencing, have been implemented to determine cDNA segments encoding variable regions of immunoglobulin (or TCRs) (1, 2). However, these low-throughput techniques lack the power to provide a broad picture of the full immune repertoire. During the past two decades, however, technical advances in high-throughput sequencing (HTS), also known as next-generation sequencing (NGS), along with evolving bioinformatic and statistical tools, have provided a new approach capable of analyzing the immune repertoire at the single sequence level. These methods create an unprecedentedly high-resolution picture of the immune repertoire and also provide massive data that cover each lymphocyte from the sample, in theory, dispensing with limitation of sequencing quantity (3).

Considering the extremely important role of the adaptive immune system in defending against infectious agents, HTS has great potential to aid in the discovery novel infectious agents and also offers new approaches for antibody or vaccine development. In this review, we introduce the implementation of HTS to the study of the immune repertoire and review the associated bioinformatic tools required for data processing and analysis. We then focus on the success of this technology in facilitating the exploration of infection-related immune repertoires for clinical diagnosis, treatment, and prevention.

## Generation of a Diverse Immune Repertoire

**Figure 1:**
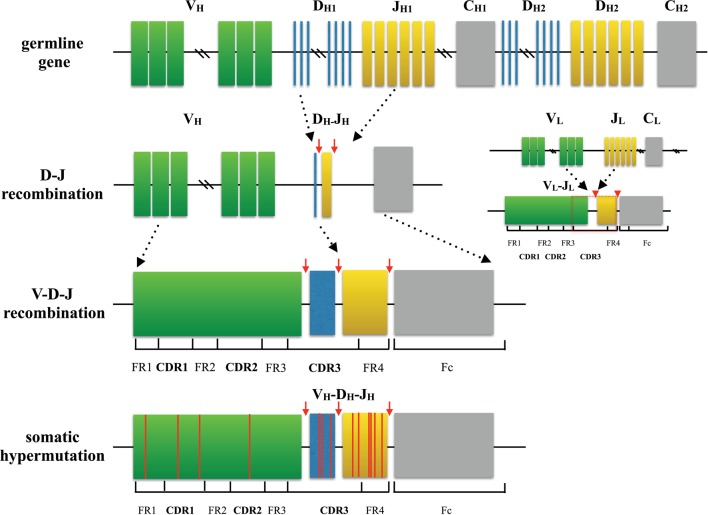
**Process of generating a diverse B cell repertoire**. The structure of each heavy chain (left) originates from rearrangement of Variable (V), Diversity (D), and Joining (J) gene segments. Recombination occurs first between D and J segment, and then V segment and D-J segment. Along with the selection of gene segments, insertion and deletion of nucleotides at the junctions between segments provides initial diversity for the primary BCR repertoire. In comparison, the light chain (right) is formed only by two segments (V and J), which makes the light chain to be less diverse. After encountering cognate antigen, somatic hypermutation introduces point mutations to frame region and complementary determining region of BCRs. This process further diversifies the repertoire and generates BCRs with higher affinity.

Amazing diversity makes the immune system the most effective system to fight against a broad scope of disease causing pathogens. This repertoire is generated by a complex series of genetic events (4). For T cells, the variable region of each TCR chain consists of three complementary determining regions (CDRs) and four frame regions (FRs). CDRs are the variable portion of the receptor and determine the antigen specificity. While CDR1 and CDR2 are formed by variable (V) gene, CDR3 is generated by random selection and recombination of variable (V), diversity (D), and joining (J) gene segments in the heavy chain (V and J region gene segments in light chain) (5, 6) (Figure 1). Thus, CDR3 is the most diverse component of a receptor, which binds MHC molecules and (or) antigens. Construction of the TCR with an alpha chain and a beta chain is also a process that contributes to receptor diversity.

The formation and revision of the T and B cell lymphocyte receptor repertoire is a highly dynamic process. The number of each lymphocyte clone changes dramatically and depends on cell specificity and the history of antigen exposure. When encountering exogenous antigens, T cells that express receptors capable of binding to a specifically compatible peptide–MHC (pMHC) complex will expand, resulting in a massive population of antigen-specific T cells that initiate the adaptive immune response (7–11). This antigen-driven proliferation process of T cells is distinct between CD4+ and CD8+ T cells after initial antigenic stimulus. Although these two types of T cells show comparable protein expression, proliferation rate, and transcriptome features after 2 days of non-infective stimulation, subsequent division of T cells differently depends on continuous existence of self-pMHC complexes. CD4+ T cells proliferate in a limited pattern, and its subsequent response requires persistent stimulation from antigen-presenting cells. CD8+ T cell is “programed” to extensive expansion after short stimulation even when transferred into antigen-free hosts (12).

The post-antigen stimulation response of B cells is more complicated because it is accompanied by somatic hypermutation and class-switch recombination that offer additional diversification of the B cell repertoire (6). Somatic hypermutation is the process of introducing point mutations at CDR1, CDR2, CDR3, and FR3 to produce B cells of higher affinity to target antigens. These higher affinity clones are then selected and expanded, which is called affinity maturation. Additionally, in class-switch recombination, the gene loci encoding the C region of BCRs are excised and replaced by a series of new constant gene segments, resulting in functional differences of IgG, IgE, or IgA that participate in different immune mechanisms during pathogen elimination.

## High-Throughput Sequencing – A New Strategy for Immune Repertoire Analysis

### Traditional Strategies for Studying the Immune Repertoire

Prior to HTS, many strategies were developed to explore post-infection immune repertoires (13). Immunoscope spectratyping has been used to investigate TCR/BCR repertoires since the 1990s (5, 14). In this technique, using one (for B cell) or more (for T cell) V or J gene specific primer pairs, the length of CDR3 can be determined (15). CDR3 length in healthy population shows a bell-shaped pattern, indicating a polyclonal repertoire. However, the unusual peaks in infected patients imply a perturbed oligoclonal repertoire with clonal expansion. As such, CDR3 spectratyping provides robust information on the complexity and stability of circulating T/B cell repertoires and insights into the immune repertoire after infection (16–20). Even if it is relatively easy and cheap, the nucleotide sequences of CDR3 remain obscure, and the extent of heterogeneity within a particular CDR3 length cannot be assessed.

Detailed nucleotide sequences of gene segments encoding the variable region can be determined by traditional DNA sequencing techniques such as Sanger sequencing (21, 22). Flow cytometry and CDR3 spectratyping help to isolate T/B cells of interest, which complements weaknesses of Sanger sequencing in quantity limitation. Single-cell sequencing is able to identify sequences of several B cells that produce monoclonal antibodies specific to certain virus, which contributes greatly to analyzing genetic features of the antibodies in the process of antibody discovery (2, 22–24). After collecting peripheral blood samples, the B cells or memory B cells are isolated and immortalized to produce antibodies. According to the HAI titers and neutralizing titers determined by ELISA, the virus-specific B cells can be identified, which helps to narrow B cell candidates for sequencing. These functional test-based antibody discovery strategies are successful but laborious. Despite this, these strategies are well designed for targeted antibody searching; however, it is insufficient for creating a high-resolution picture of the human immune repertoire.

### High-Throughput Sequencing of Lymphocyte Repertoires

High-throughput sequencing has recently become a novel and powerful tool to investigate the immune repertoire. The depth and comprehensiveness of high-throughput immune repertoire sequencing are greater than ever, and the enormous sequencing data of disease-specific TCR/BCR clones provide great potential for the revealing dynamic changes in clonality during infectious states.

Establishing a lymphocyte repertoire database starts from sample collection from carefully selected populations and isolation of interested T cell or B cell subgroups. Due to the well-acknowledged heterogeneity of TCRs and BCRs between individuals, longitudinal studies tracking dynamic alterations in certain population help to reduce difficulties in data interpretation at unraveling the infection course. Classification of subgroups of T cell and B cells, e.g., naive and memory T/B cells, CD4+, and CD8+ T cells, is necessary if distinct behavior of these subgroups is considered in detail.

The methodology of library preparation and amplification need careful design since it affects accuracy of the ultimate sequencing data. Due to the difference of V and J gene segments, a common primer does not apply to sequencing of CDR3. Multiplex PCR is capable to amplify multiple loci simultaneously and, however, is likely to introduce bias. It is because of non-specific amplification, primer-dimer formation, and uneven reaction conditions. More precise and quantitative multiplex PCR may be achieved through primer concentration adjustment and bias filtering using amplification bias among the templates as controls (23). Another alternative PCR method is 5′RACE PCR, which provides a less biased PCR library using primers that bind downstream of the variable domain (24).

Sequencing techniques are evolving continuously to be deeper and more precise, and there are three prevalent HTS platforms available today. The comparison of mechanisms, sequencing depth, and other critical features of each platform is shown in Table 1. The Illumina and Roche 454 platforms have been most commonly used during immune repertoire analysis. The outputs of each platform must be analyzed with caution because of notable platform-specific sequencing error (25–28). Insertions and deletions of nucleotides, resulting from imperfect interpretation of homopolymeric stretches, are considerable for the Roche 454 platform (29), while substitution errors are predominant in Illumina platform (30, 31). The overall error rate of Illumina platform is lowest while that of Ion Torrent is highest among the three (32). In an attempt to correct sequencing errors, three algorithms are most commonly used including k-mer spectrum, multiple sequence alignment, and suffix tree (26). Based on these algorithms, bioinformatic tools are designed for different platforms, for example, BFC (33), HiTEC (34), Lighter (35), Reptile (36), and ECHO (37) are for Illumina platforms, and PyroNoise (38), DeNoiser (39), and HECTOR (40) are for 454 platforms. Their main approaches, correction functions, and qualities are compared in Ref. (27).

**Table 1:** **High-throughput sequencing platforms used for Lymphocyte Repertoire Study**.

HTS platform	Mechanism	Read depth	Read length	Typical throughput	Covered region	Application	Accuracy
Roche 454 GS FLX Titanium XLR70	Pyro-sequencing	~1 million	Up to 600 bp	450 Mb	FR1-C region	BCR (long read), TCR (long read)	Consensus accuracy 99.995%
Illumina HiSeq	Dye terminator sequencing	~2 billion	2 × 250 bp	500–1000 Gb	FR3-C region	TCR (CDR3), BCR (long read), TCR (long read)	>80% bases above Q30
Illumina MiSeq	Dye terminator sequencing	~25 million	2 × 150 bp	13.2–15 Gb	FR1-C region	TCR (CDR3)	>80% bases higher than Q30 at 2 × 150 bp
			2 × 300 bp				>75% bases higher than Q30 at 2 × 250 bp
Ion Torrent/Life Technologies	Semiconductor sequencing	~1 billion	Up to 200 bp	30 Mb–2 Gb	FR3-C region	TCR	>97% bases

PCR and sequencing errors inevitably result in overestimate of repertoire diversity. The common statistical strategy for both PCR and sequencing error removal is eliminating low abundance and low-quality sequences (with low Phred score), but it leads to a great loss of sequencing information. To rescue these sequences, low-quality CDR3 sequences with no more than three low-quality nucleotides can be mapped to “core clonotypes” derived from high-quality sequences with allowed mismatches at low-quality position. Then, the low-abundance core clonotypes are merged with the high-abundance core clonotypes with less than three allowed mismatches at V (≤2 mismatches), D (≤1 mismatches), and J (≤2 mismatches) gene segments to correct PCR errors. This integrated algorithm based on sequence quality abundance could efficiently correct artificial errors and avoid information loss, thus providing more reliable estimation of repertoire diversity (28). Unique molecular identifiers that label each starting molecule help to reduce both PCR and sequencing errors (24, 41). Combined with this experimental strategy, molecular identifier groups-based error correction (MIGEC) corrects PCR and sequencing errors more efficiently than other quality- and frequency-based strategies (42).

Determination of the V–D–J gene segments from which the CDR3s are rearranged, as well as identification of point mutations, is often achieved using the ImMunoGeneTics database (http://www.imgt.org) (43), despite the controversies about its validity (44–46). New V–D–J gene annotation tools based on various algorithms are reported, such as IgBLAST (47), iHMMune-align (48), and Decombinator (49, 50) (Table 2). In addition, many integrated bioinformatic tools (MiTCR, LymAnalyzer, Change-O, etc.) for data processing are developed recently (51–62) (Table 2), which provides various statistical approaches for diversity estimation, repertoire comparison, clustering analysis, and somatic hypermutation analysis (Table 3). Despite these tools, standardized bioinformatic analysis and visualization strategy is lacking, which remains the main obstacle for comparison of researches from different investigators.

**Table 2:** **Annotation and integrated bioinformatic tools for Lymphocyte Repertoire Data Analysis**.

Bioinformatic tools	Models	Key feature	Limitation	Cases of infection study	Reference
**For VDJ gene assignment**					
IMGT/HighV-QUEST	–	Designed for NGS data; most commonly used	Online version; results sent to user after 1–2 weeks	MERS-CoV (63), influenza (64)	(65)
iHMMune-Align	Hidden Markov model (HMM)	Most probable gene segment; accurate IGHD gene identification	Nucleotide insertions or deletions at the gene junctions but not within germline genes	Influenza vaccine (66); EBV, CMV (67)	(48)
SoDA2	Hidden Markov model (HMM)	Probability model; ≥1 probable rearrangement candidates	–	HIV (68)	(50)
IgBlast	BLAST algorithm	Open source; gene databases search simultaneously	Relatively low throughput (<1000 per batch)	Dengue virus (69); HIV (70)	(47)
Decombinator	Aho–Corasick algorithm	Increased speed	Better matches between query sequence and target sequence needed; only for TCR	*M. tuberculosis* (71) HIV (72)	(49)
**Integrated toolkits**					
O-Change	Integrated	Functions unique to BCR analysis (somatic hypermutation analysis, quantifying selection pressure, and calculating sequence chemical properties)	–	West Nile Virus (73)	(54)
LymAnalyzer	Integrated	Accurate and complete assignment; polymorphism and SHM analysis	–	–	(56)
tcR	Integrated	Based on R; diversity calculation; repertoire comparison; visualization	For output of MiTCR	–	(57)
iMonitor	Integrated	Re-alignment to improve accuracy; visualization; PCR and sequencing errors correction	No polymorphism and SHM analysis	–	(59)
MiTCR	Integrated	Accurate and higher speed; PCR and sequencing errors correction; sequence quality filter; low quality sequencing data rescue	Strongly related to sequencing quality; only for TCR	CMV (74)	(58)
ImmunExplorer (IMEX)	Integrated	Simple statistical analysis and visualization; repertoire comparison	For IMGT/HighV-QUEST outputs	–	(55)
VDJtools	Integrated	Diversity assessment, clustering analysis, CDR3 region analysis, and data visualization; simple and user-friendly	Only for TCR; compatible annotation tools needed	–	(61)
sciReptor	Integrated	Single-cell sequencing data and HTS data; supports flow cytometry index data; clustering and SHM analysis	–	–	(60)
**Others**					
Tool for Ig genotype elucidation *via* Rep-Seq (TIgGER)		Novel V alleles identification; personalized germline database construction	Complementary tools for IMGT; only for novel alleles not distantly related; only for TCR		(75)

**Table 3:** **Statistical algorithms and models for Lymphocyte Repertoire Data Analysis**.

Statistical methods	Functions	Reference
Maximum entropy models	Statistical properties of the repertoire	(76)
Capture–recapture analysis	Diversity assessment	(77, 78)
Shannon index	Diversity assessment	(79)
Gini–Simpson index	Diversity assessment	(79, 80)
Chao1 algorithm	Richness (maximum number of clones)	(81)
Abundance-based coverage estimator	Richness (maximum number of clones)	(82)
Chao2	Lower bound of species richness	(83) (84)
Wu–Kabat index		
Poisson abundance model	Diversity and clone distribution estimation	(85, 86)
Morisita–Horn similarity index	Repertoire similarity comparison	(87)
Clonality score	Convergence-level estimation	(67, 84, 88)
Hidden Markov model	V and J assignment (probability based)	(48, 50)
Aho–Corasick algorithm	V and J assignment	(49)
Fast-tag-searching algorithm	V and J assignment (Hamming distance based)	(56)
Single nucleotide polymorphism (SNP) calling algorithm	Identify novel VDJ gene alleles	(89)

## Progress in Infection-Related Immune Repertoire

High-throughput sequencing techniques have revolutionized the study of the immune repertoire. Utilizing HTS, many important insights into mechanisms of immune response have been gained. It is also the cornerstone for potential clinical applications of repertoire analysis, including identification of diagnostic biomarkers, design of therapeutic antibodies, and development of new vaccines.

### Assessing Dynamic Changes in the Immune Repertoire after Antigen Stimulation

Estimating the diversity of a TCR/BCR repertoire is necessary for estimating the theoretical size of the repertoire and for tracking changes in clonal populations during the clinical course of infection. Several different methods may be used to describe the diversity of lymphocyte repertoires at different levels – VDJ recombination diversity (90), Simpson diversity index (91, 92), and some non-parametric methods. Decrease in the overall diversity of the immune repertoire have been observed after various antigen exposures, including HIV, influenza, and human herpes virus, which implies expansion of particular T/B cell clones (67, 88, 92, 93). Our group compared changes in the diversity of the TCR beta chain and BCR heavy chain after H7N9 virus infection. Interestingly, these results show that the diversity of the BCR heavy chain starts to increase 2 weeks after H7N9 infection, while the TCR beta chain repertoire continues to contract. In addition, a more diverse BCR repertoire and a less diverse TCR beta chain repertoire in convalescent phase correlate with improved prognoses, implying differences in the response process of humoral and cellular immunity.

The immune response to vaccination has been used as an ideal model for antibody repertoire research due to the convenience drawing blood samples at well-defined time points (94). Studies using vaccines, such as influenza and TT, have revealed dynamic changes in the size and diversity of antibody repertoires before and after antigen stimulation (41, 90, 95, 96). Comparison of post-vaccination responses suggests divergent repertoire properties among individuals, different age groups, and successive immunization of the same individual with different influenza vaccines (TIV and LAIV) (66, 90, 95). The maximum clonal response has been found to occur 7 days after vaccination, but the magnitude of response varies between individuals despite an identical immune challenge, which may be influenced by previous exposure, age, and other concurrent immune responses. In addition, study of B cell memory can be achieved by repeated sequencing of samples taken from the same individual in separate immune responses to an antigen. Vollmers and colleagues identified a group of B-cell clones as a recall response to two substantially different vaccine compositions, implying the possibility of identifying cross-specific antibody using repertoire analysis (41).

Immune responses recorded by sequencing data have also been useful in testing the role of adjuvants in eliciting broad-spectrum antibodies. Wiley and co-workers tested the immune response of mice immunized with malaria vaccine by analyzing IgG repertoires. They found that TLR agonist used as adjuvant increases the diversity of IgG variable region, which is related to improved ability of the antibodies to recognize a broad spectrum of epitopes (97). These studies exemplify a new level of details assessing vaccine response and pioneered HTS implications in vaccine design.

### Signatures of TCR/BCR Sequences for the Diagnosis of Infectious Diseases

In infected patients, antigen-specific T/B cell repertoires form in response to antigen exposure in both circulation and peripheral tissues. Immune repertoire sequencing provides broad information including crucial antigen-specific clones, which have the potential to halt the spread of pathogens (98). Diagnostic marker discovery using sequencing data relies on these antigen-specific clones with stereotyped features in the post-infection repertoires. These features are assessed at different levels such as gene rearrangement, identical or similar CDR3 sequence overlap, and certain CDR3s length.

After influenza H1N1 vaccination, the dominant clonotype of Ig heavy chains has the same V–J gene rearrangement, CDR3 length, and somatic mutation position in CDR1 and CDR3 with previously reported influenza antibodies (66). However, in this study, the convergent dominant sequence is only found in one individual. Further researches in a broader population including non-dominant sequences are needed. A more successful example is reported in Ig repertoires related to dengue virus infection. Using cross validation and other approaches, stereotyped CDR3 sequences or CDR3 lengths that have high prevalence in the acute dengue samples are found to be specific to acute dengue infection, which are absent or of low prevalence in healthy and post-convalescent population (88).

Identification of pathogen-specific sequences also helps in differential diagnosis between infectious and non-infectious diseases. According to Dziubianau et al., comparing PBMCs-derived T cell clonotypes specific to a given virus with T cells from different origins (allograft-derived and urine-derived lymphocytes) provides a new methodology for differential diagnosis of two post-transplant complications – BKV-associated nephropathy and acute cellular rejection, which shows a glimpse of applications of T cell sequencing in diagnosis (99). In addition, a recent study searched sequencing data for CDR3 amino acid motifs that have been reported to be specific for a particular pathogen and succeeded in identifying CDR3 sequences identical or similar to these motifs in post-vaccination volunteers (100). According to these results, interestingly, it is low frequency sequences that possess the probability of becoming promising biomarkers instead of the dominant ones.

However, immune responses show dramatic differences in CDR3 sequences responding to same pathogens across individuals and age groups. This intrinsic divergence between individuals is the major obstacle in finding “public” sequences as optimal biomarkers. Nevertheless, we hold a promise for the application of HTS data in differential diagnosis because it provides a large number of candidate sequences for biomarker investigation. Instead of using single biomarkers such as PSA or AFP in diagnosis, a combination panel of selected sequences may establish a pathogen-specific sequence library for diagnosis, which holds the potential of unprecedented sensitivity and specificity.

### Identification of Antigen-Specific Antibodies and T Cells Based on High-Throughput Sequencing Data

Recombinant monoclonal antibodies have great potential in the treatment of specific infections. In recent years, several strategies, including phage display libraries, single B cell expression, and B cell immortalization, have been used to discover antibodies against specific antigens (101). HTS of the antibody repertoire, combined with subsequent bioinformatic tools or traditional screening tools, has facilitated the identification of antigen-specific sequences (102–105).

The methodology predicting antigen specificity completely from analysis of BCR sequences has not been possible yet, albeit it has considerable potential in immune repertoire studies. Nevertheless, there have been many efforts made in mining the HTS data for functional antibodies (106). A relatively direct method for identifying such sequences is based on the similarity of amino acid sequence to previously reported antibodies. Researchers have successfully found sequences of high identity with the broadly neutralizing antibodies and strain-specific antibodies from established antibody repertoires of patients with influenza infection or vaccination (66). Some of these sequences have proven to have neutralizing activity, validating the potential of deep sequencing-based antibody identification. Success of this work also suggests the possibility of monoclonal antibody synthesis without cell cloning for treatment (66). Furthermore, another method using the frequency rank of heavy chain and light chain sequences to predict the function of antibody sequences has been reported successful in mouse models (107).

Exciting work by Kwong and coworkers demonstrates the feasibility of identifying neutralizing active clones through bioinformatic analysis from HIV patients (108, 109). They established several steps for interrogating variants of known neutralizing antibody classes from HIV-infected patients, with or without previous knowledge if the patient had antibodies belonging to this family. First, the heavy or light chain sequences, derived from the germline IGHV or IGLV gene same as template antibody, are isolated from a new donor. Then, these sequences were compared with the germline gene for “divergence” and the template antibody sequence for “identity,” generating a contour plot called “divergence/identity plot.” The sequences segregate into clusters in the plot, from which the high divergence and high identity sequences were selected as candidates for neutralizing antibodies. This process is called “Grid-based strategy.” Then, the germline sequence, candidate sequences, and template antibody sequences of the same class are merged to build a phylogenetic tree rooted by the template sequence, which is called “cross-donor analysis.” The heavy chain sequences from new donor clustered in the subtree of the template sequences are then expressed with template light chain to generate antibodies. Most of these sequences have neutralizing activity to HIV-1. The outstanding efficiency of this method was also demonstrated when compared to sequence-based strategy and prevalence-based strategy (108, 109). Lu et al. also validated this phylogenetic method in identifying functional anti-*Staphylococcus aureus* antibodies (110). These strategies start from previously reported antibody sequences. However, such antibody sequences are not always available, especially during poorly characterized viral infections such as H7N9.

Pairing the heavy and light chains as an integrated antibody has been another challenge for HTS-based immune repertoire analysis. In most cases, researchers only focus on the heavy chain, which causes a critical loss of antibody integrity and leads to problems in following synthesis of artificial monoclonal antibody. Two strategies have been reported to correctly pair the heavy chain and light chain sequences based on the frequency or evolution models. Reddy and colleagues (107) have pioneered pairing based on the frequency ranks, using plasma cells isolated from bone marrow of immunized mice and matching the two chains of similar rank order. Monoclonal antibodies expressed in this way did show antigen specificity. Due to the linkage of heavy chain and light chain as an integrated protein, their evolution undergoes the same enzymatic mutation process, and they evolve together to bind the same antigen with high affinity. Based on this theoretical foundation, phylogenic analysis has been used as another method to compare the evolutionary topography of the heavy chain and light chain after bioinformatic identification of transcripts related to a known HIV neutralizing antibody (109, 111). Reconstituted novel antibodies consist of phylogenetically matched chains showing similar neutralizing function but less auto-reactivity compared to the mismatched ones.

Several groups have recently achieved advances in the technology of paired sequencing of antibodies. Single-cell PCR has been utilized to create a two-dimensional bar-coded primer matrix to link two chains of the BCR (112). Using this technique, Busse and coworkers analyzed paired sequences of over 46,000 B cells in one experiment and accomplished subsequent antibody gene cloning and expression. At the same time, Turchaninova and coworkers performed pioneering research in emulsion-based technology for sequencing antibody repertoires of paired chains (113). They used water-in-oil emulsions for cell-based overlap expansion RT-PCR, although its yield was relatively low yield. Another high-throughput paired sequencing method by DeKosky et al. used micro well plates to isolate B cells and magnetic beads to capture mRNAs (114). Very recently, DeKosky’s group combined and improved these previous techniques, and developed a cost-effective and efficient methodology to establish a more precisely paired repertoire (115).

Predicting T cell specificity based on TCR heterodimer sequence is more difficult than antibodies because of the highly variable nature of each of the components of the TCR–peptide–MHC complex (116). Due to the challenges posed by the highly variable CDR3 loop of the TCR and the complexity of predicting protein–protein interactions (117, 118), experimental functional tests for mining antigen-specific T cells might be a more fruitful approach (119).

### Implementation of Immune Repertoire Analysis in Vaccine Development

Recent advances in HTS-based antibody sequencing may provide the biggest benefit for the field of vaccine development. Over the years, efforts to elicit protective immune responses to HIV by immunization have not been successful. During acute viral infections, high-affinity neutralizing antibodies develop in just weeks. However, generating effective broadly neutralizing antibodies during chronic infections, such as HIV, takes significantly longer time. Furthermore, the neutralizing power of these antibodies is often variable due to impairment of the host immune function, unusual features of Env, and co-evolution of the virus in response to the host antibody response (120, 121).

Deep sequencing analysis has identified rare variants of known HIV-neutralizing antibodies and has elucidated the ontogeny of these neutralizing antibodies (108, 109, 111, 122). These findings have cast a light on antibody guided vaccine development. In following studies, the HTS-based phylogenetic strategy greatly facilitated study in co-evolution of neutralizing antibodies and virus mutants (123). Combined with long-term follow-up studies, these results illustrate how mutations in some sites allow the virus to escape some neutralizing antibodies, and how the virus, with the help of secondary neutralizing antibodies, becomes sensitive to the neutralizing antibody (98, 124, 125). These studies suggest a promising pathway to elicit broadly neutralizing antibodies by sequential immunization with selected immunogens (123, 126). Furthermore, structure studies of the neutralizing antibody family provide candidates for future vaccine designs (127).

## Conclusion

High-throughput sequencing has been a breakthrough technology for the study of the immune repertoire and has already had a profound effect on our knowledge of the immune systems physiology during health and disease. In particular, HTS has transformed our understanding of immune repertoire formation during infection, malignancy, and autoimmunity. Advances in this filed of research will rely on progress of similar laboratory techniques. Collection of more precise sequencing data can be anticipated with consistent improvement of sequencing techniques and error correction strategies. An increasing number of researchers have shown interest in this area in recent years, and this has provided vast quantities of data that could provide answers to important existing questions. These data repositories should be effectively utilized. Establishing a public database and collecting deep sequencing data for collective collaboration will facilitate information exchange and the investigation of the varieties of repertoires across gender, age, race, and healthy state. In addition, development of standardized bioinformatic tools will be indispensable for harnessing HTS output.

Although the expected potential of immune repertoire studies in clinical use is enormous, more work remains to be done to incorporate the observed dynamic changes and sequence signatures with clinical features and outcomes. Questions remain about how the severity of certain infections is related to alterations of the immune repertoire response and various manifestations of CDR3 sequences, and how to predict abundance of protective immunoglobulins or T cell from a given sequence library. In terms of therapeutic discoveries, identification and production of functional antibodies and T cells will promote the development of passive immune therapies and vaccines. Traditional and recently reported large-scale screening strategies may contribute greatly to this process. Advances in HTS of the immune repertoire during health and disease will provide comprehensive views of the adaptive immune response in very near future and will open the door to more rationale immunotherapy for infection.

## Author Contributions

DH was involved in study design, wrote the first draft of the manuscript, conducted the literature search, reviewed the abstracts, performed the analysis, and contributed to the final draft; CC and SC were involved in study design and reviewed the abstracts. ES revised the manuscript. YS designed and supervised the study, revised the final draft, and contributed to the analysis. All authors have read and approved the final manuscript.

## Conflict of Interest Statement

We have no financial or personal relationships with other people or organizations that can inappropriately influence our work; there is no professional or other personal interest of any nature or kind in any product, service, and/or company that could be construed as influencing the position presented in this article.
